# Blunt Traumatic Aortic Injury: 10-Year Single-Center Experience

**DOI:** 10.1055/s-0040-1715608

**Published:** 2021-03-24

**Authors:** Ahmet Can Topcu, Kamile Ozeren-Topcu, Ahmet Bolukcu, Sinan Sahin, Avni U. Seyhan, Ilyas Kayacioglu

**Affiliations:** 1 Department of Cardiovascular Surgery, Dr. Siyami Ersek Thoracic and Cardiovascular Surgery Training and Research Hospital, Istanbul, Turkey; 2 Department of Radiology, Dr. Siyami Ersek Thoracic and Cardiovascular Surgery Training and Research Hospital, Istanbul, Turkey; 3 Department of Emergency Medicine, Kartal Dr. Lutfi Kirdar Training and Research Hospital, Istanbul, Turkey

**Keywords:** aorta, endovascular, surgery, thoracic, trauma

## Abstract

**Objective** In blunt trauma patients, injury of the thoracic aorta is the second most common cause of death after head injury. In recent years, thoracic endovascular aortic repair (TEVAR) has largely replaced open repair as the primary treatment modality, and delayed repair of stable aortic injuries has been shown to improve mortality. In light of these major advancements, we present a 10-year institutional experience from a tertiary cardiovascular surgery center.

**Methods** Records of patients who underwent endovascular or open repair of the ascending, arch or descending thoracic aorta between January 2009 and December 2018 were retrospectively analyzed. Patients without blunt traumatic etiology were excluded. Perioperative data were retrospectively collected from patient charts. Long-term follow-up was performed via data from follow-up visits and phone calls.

**Results** A total of 1,667 patients underwent 1,740 thoracic aortic procedures (172 TEVAR and 1,568 open repair). There were 13 patients (12 males) with a diagnosis of blunt thoracic aortic injury. Mean patient age was 43.6 years (range, 16–80 years). Ten (77%) patients underwent TEVAR, two (15.4%) underwent open repair, and one (7.7%) was treated nonoperatively. Procedure-related stroke was observed in one (7.7%) case. Procedure-related paraplegia did not occur in any patients. Left subclavian artery origin was covered in seven patients. None developed arm ischemia. Hospital survivors were followed-up for an average of 60.2 months (range, 4–115 months) without any late mortality, endoleak, stent migration, arm ischemia, or reintervention.

**Conclusion** Blunt thoracic aortic injury is a rare but highly fatal condition. TEVAR offers good early and midterm results. Left subclavian artery coverage can be performed without major complications.

## Introduction

In blunt trauma patients, injury of the thoracic aorta is the second most common cause of death after head injury. 1 2 Up to 85% of patients with blunt thoracic aortic injury (BTAI) die at the scene or during transfer to a medical facility. 1 2 3 Of those who reach hospital alive, 50% die before aortic repair can be performed. 2

The landmark study by Parmley and colleagues 4 in 1958 demonstrated the high mortality associated with BTAI. Since then, management of patients with BTAI has gone through some major advancements. With widespread availability of multidetector scanners, computed tomography angiogram (CTA) has replaced plain chest X-ray as a screening tool and aortogram as a diagnostic tool. 5 6 Following the first report of endovascular treatment of BTAI with custom devices, thoracic endovascular aortic repair (TEVAR) has largely replaced open repair as the primary treatment modality. 5 6 7 8 Delayed repair of stable aortic injuries has been shown to improve mortality. 5 6 9 In light of these major advancements in the diagnosis and management of BTAI, we present 10-year institutional experience from a tertiary cardiovascular surgery center.

## Materials and Methods

### Patients

The study design was approved by Institutional Research Ethics Committee. All procedures related to the study were conducted in accordance with the ethical standards of the Helsinki Declaration. Individual consent was waived due to the retrospective nature of the study. Records of patients who underwent endovascular or open repair of the ascending, arch or descending thoracic aorta between January 2009 and December 2018 were retrospectively analyzed. A total of 1,667 patients underwent 1,740 thoracic aortic procedures (172 TEVAR and 1,568 open repair). Patients without blunt traumatic etiology were excluded. All trauma patients were referred with a diagnosis of BTAI from other medical facilities for cardiovascular intervention. Perioperative data were retrospectively collected from patient charts.

### Initial Management

Patients were admitted to a dedicated cardiovascular surgery intensive care unit (ICU). Meticulous intravenous fluid replacement was administered with care to avoid “hyperresuscitation.” Intravenous esmolol infusion was used to maintain systolic blood pressure below 120 mm Hg. Where esmolol was insufficient, intravenous nitrates were started.

### Imaging

**Fig. 1:**
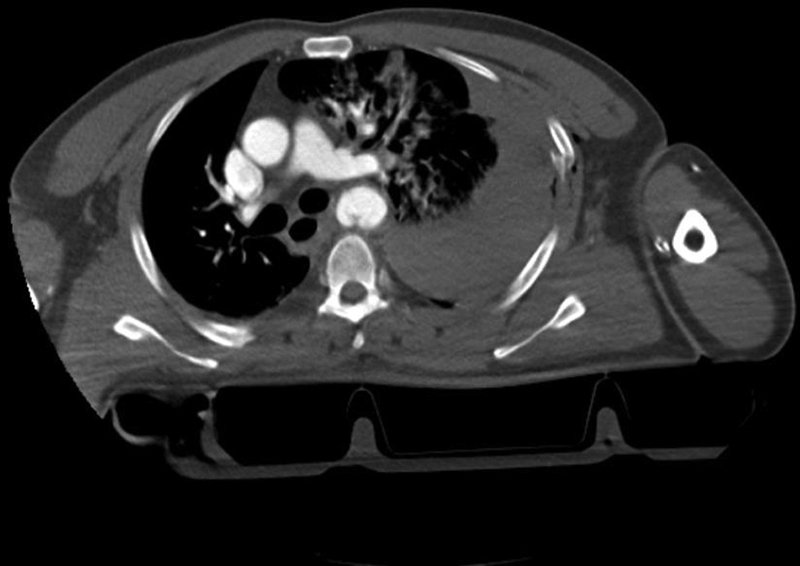
Computed tomography angiogram demonstrating aortic isthmus pseudoaneurysm, mediastinal hematoma and left hemothorax after blunt trauma.

CTA of the chest, head, and neck was used for aortic measurements, injury grading, and preoperative planning ( Fig. 1 ). Severity of aortic injury was classified according to clinical practice guidelines of the Society for Vascular Surgery (SVS): grade I, intimal tear; grade II, intramural hematoma; grade III, aortic pseudoaneurysm; and grade IV, free rupture. 10 11 Cerebral vascular anatomy was assessed for possible left subclavian artery (LSA) coverage.

### Intervention

Patients underwent TEVAR if they were suitable candidates. The decision between TEVAR and open repair was made based on anatomical location of aortic injury, patient's vascular anatomy, concomitant injuries, and hemodynamics, and availability of an endovascular device.

Endovascular procedures were performed in a dedicated catheterization laboratory under general anesthesia. Vascular access was gained using open femoral artery exposure. Routine arch aortogram was performed. Patients received low-dose systemic heparin. LSA origin was covered if necessary to obtain adequate proximal seal. Protamine was given for heparin reversal.

Patients unsuitable for TEVAR underwent open repair. Operations were performed under general anesthesia using extracorporeal circulation through sternotomy, left thoracotomy, or sternothoracotomy with full-dose heparin. Cerebrospinal fluid drainage was used where necessary. Protamine was given for anticoagulation reversal.

### Outcome and Follow-up

**Fig. 2:**
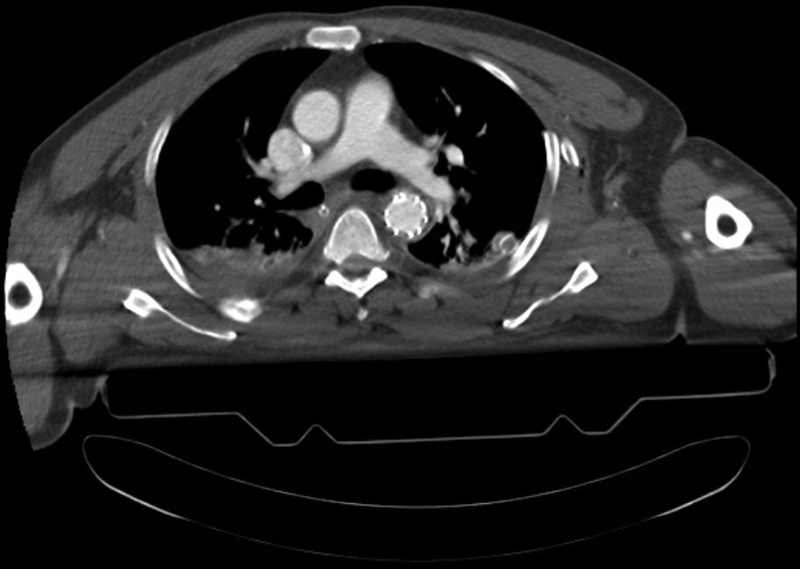
Follow-up computed tomography angiogram after endovascular repair for blunt thoracic aortic injury.

All survivors returned to ICU. Following cardiovascular stabilization, patients were either discharged home or referred to other facilities for treatment of their concomitant injuries. Patients who underwent TEVAR were followed up with CTA at 1, 6, and 12 months after procedure ( Fig. 2 ). Long-term follow-up was performed via data from follow-up visits and phone calls.

## Results

Thirteen patients (12 males) with a diagnosis of BTAI were treated in our institution between January 2009 and December 2018. Mean patient age was 43.6 years (range, 16–80 years). The most common mechanism of injury was motor vehicle collision ( *n* = 5, 38.5%), followed by fall from height ( *n* = 4, 30.8%). Hypotension (systolic blood pressure ≤ 90 mm Hg) was present in four (30.8%) patients. Five (38.5%) patients had Glasgow coma scale (GCS)< 15 on admission. Mean injury severity score (ISS) was 32.3 (range, 16–48; Table 1 ).

**Table 1:** Patient demographics

Characteristic	*N*	%
Age (y) Mean (range)	43.6 (16–80)	
Male	12	92.3
*Trauma mechanism:*		
Motor vehicle collision	5	38.5
Fall from height	4	30.8
Motorcycle collision	2	15.4
Pedestrian hit by vehicle	1	7.7
Crushed under falling object	1	7.7
Hypotension on admission (SBP ≤ 90 mm Hg)	4	30.8
GCS < 15 on admission	5	38.5
GCS Mean (range)	11.8 (3–15)	
ISS Mean (range)	32.3 (16–48)	

Abbreviations: GCS, Glasgow coma scale; ISS, injury severity score; SBP, systolic blood pressure.

Nine (69.2%) patients underwent TEVAR, two (15.4%) underwent open repair and one (7.7%) underwent TEVAR followed by open repair ( Table 2 ). One patient was managed nonoperativelly, and eventually developed a chronic pseudoaneurysm at isthmus level and a juxtarenal coarctation caused by abdominal surgery for liver injury. This patient was treated with a successful hybrid procedure 5 years after the index trauma. LSA origin was covered in seven patients. None developed left arm ischemia. Excluding one patient with ascending aortic injury, mean diameter of the healthy aorta proximal to injury was 26.7 mm (range, 19–36.8 mm). Mean stent diameter and length were 27.4 mm (range, 22–36 mm) and 124 mm (range, 80–150 mm), respectively. Mean interval between admission and intervention was 33.7 hours (range, 3.5–95.7 hours). Six patients underwent delayed repair (repair > 24 hours; Table 2 ).

**Table 2:** Operative details

Characteristic	*N*	%
*Type of aortic intervention:*		
TEVAR	9	69.2
Open repair	2	15.4
TEVAR followed by open repair	1	7.7
NOM	1	7.7
*LSA coverage:*	7	–
Aortic diameter proximal to injury (mm) Mean (range) a	26.7 (19–36.8)	
Stent diameter (mm) Mean (range)	27.4 (22–36)	
Stent length (mm) Mean (range)	124 (80–150)	
*Surgical incision:*		
Thoracotomy	1	–
Sternotomy	1	–
Sternothoracotomy	1	–
*Perfusion technique:*		
Total CPB	2	–
Left heart bypass	1	–
Interval between admission and intervention (h) Mean (range)	33.7 (3.5–95.7)	
Delayed repair (>24 hours)	6	–

Abbreviations: CPB, cardiopulmonary bypass; LSA, left subclavian artery; NOM, nonoperative management; TEVAR, thoracic endovascular aortic repair.

a Excluding one patient with ascending aortic injury.

There were two (15.4%) in-hospital deaths. One death was aortic related, and the other was caused by sepsis. Among hospital survivors, mean duration of mechanical ventilation was 257.8 hours (range, 2–2112 hours), mean ICU length of stay (LOS) was 15.6 days (range, 1–89 days), and mean hospital LOS was 29.3 days (range, 2–105 days). One patient experienced post-TEVAR aortic rupture, and was successfully treated with replacement of the descending aorta through a left posterolateral thoracotomy using left heart bypass. There were three (23.1%) patients with stroke and three (23.1%) with paraplegia. Stroke was procedure-related in one (7.7%). Other stroke cases were due to concomitant head injuries. All three patients with paraplegia had concomitant spinal cord and/or thoracolumbar spine injury, and were paraplegic on admission. Hospital survivors were followed-up for an average of 60.2 months (range, 4–115 months) without any late mortality, endoleak, stent migration, arm ischemia, or reintervention ( Table 3 ).

**Table 3:** Postoperative course

Detail	*N*	%
*In-hospital mortality:*	2	15.4
Aortic-related mortality	1	7.7
Duration of mechanical ventilation (hours) Mean (range) a	257.8 (2–2112)	
Intensive care unitlength of stay (d) Mean (range) a	15.6 (1–89)	
Hospital length-of-stay (d) Mean (range) a	29.3 (2–105)	
Follow-up duration (mo) Mean (range) a	60.2 (4–115)	
*Complications:*		
Stroke	3	23.1
Procedure-related	1	7.7
Paraplegia	3	23.1
Procedure-related	0	0
Renal failure	2	15.4
Sepsis	3	23.1
Pneumonia	3	23.1
Intra-abdominal infection	1	7.7
Urinary tract infection	1	7.7
Wound infection	1	7.7
Delirium	1	7.7

a Excluding patients who did not survive to hospital discharge.

**Fig. 3:**
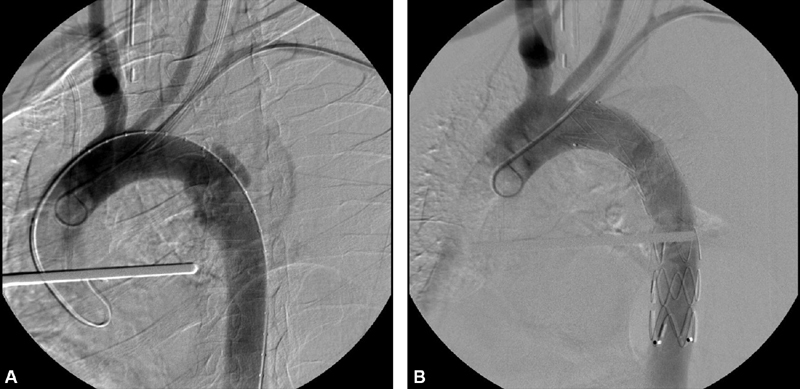
( **A** ) Arch aortogram demonstrating aortic isthmus pseudoaneurysm; ( **B** ) successful endovascular repair.

Diagnosis was made by means of CTA in all patients. Aortic isthmus was the most common site of injury ( *n* = 12; Fig. 3A and B ). There were two (15.4%) SVS grade-IV injuries, four (30.8%) grade-III injuries, six (46.2%) grade-II injuries, and one (7.7%) grade-I injury ( Table 4 ). Two patients with grade-IV aortic injuries presented in extremis with cardiac tamponade. They both underwent emergency open repair with total CPB. One of them had a free rupture of the proximal ascending aorta, and underwent a Bentall's procedure with mechanical valved conduit through a sternotomy. He had an underlying ascending aortic aneurysm with a diameter of 55 mm. Although his recovery was complicated by an ischemic stroke, this patient survived to discharge and is symptom free during a 5-year follow-up period. The other patient with grade-IV injury underwent replacement of the descending aorta through a clam shell incision, and did not survive.

**Table 4:** Aortic injury characteristics

Characteristic	*n*	%
*Diagnostic modality:*		
Computed tomography angiogram	13	100
*Aortic injury location:*		
Isthmus	12	92.3
Ascending aorta	1	7.7
*Society for Vascular Surgery grade:*		
I	1	7.7
II	6	46.2
III	4	30.8
IV	2	15.4

Chest X-rays revealed mediastinal widening in all but one patient ( *n* = 12). There was hemothorax in ten (77%) patients, multiple rib fractures in seven (53.8%), abdominal/pelvic organ injury in seven (53.8%), and long bone fractures in six (46.2%; Table 5 ). None of the patients received craniotomy or maxillofacial reconstruction. A chest tube was inserted in ten (77%) patients. Orthopaedic operations were performed in four (30.8%) patients, laparotomy in three (23.1%), and spinal surgery in one (7.7%; Table 6 ).

**Table 5:** Concomitant injuries

Injury type	*n*	%
Head injury	3	23.1
Maxillofacial injury	3	23.1
Multiple rib fractures	7	53.8
Flail chest	1	7.7
Mediastinal widening	12	92.3
Hemothorax	10	77
Pneumothorax	5	38.5
Pulmonary contusion	5	38.5
Blunt myocardial injury	1	7.7
Cardiac tamponade	2	15.4
Diaphragm rupture	1	7.7
Abdominal/pelvic organ injury	7	53.8
Spinal cord injury	2	15.4
Long bone fracture	6	46.2
Sternal fracture	1	7.7
Pelvic fracture	5	38.5
Cervical spine injury	1	7.7
Thoracic spine injury	4	30.8
Lumbar spine injury	3	23.1

**Table 6:** Additional interventions

Intervention	*n*	%
Craniotomy	0	0
Maxillofacial reconstruction	0	0
Chest tube insertion	10	77
Laparotomy	3	23.1
Spinal surgery	1	7.7
Orthopaedic surgery	4	30.8

## Discussion

A retrospective analysis of the National Trauma Data Bank revealed that incidence of BTAI among all trauma admissions is 0.3%. 12 However, autopsy studies report thoracic aortic injuries to be present in 17 to 34% of blunt traumatic fatalities. 13 14 The discrepancies between clinical and autopsy series are due to high mortality of BTAI. In fact, 75 to 85% of patients die before reaching a medical facility, and of those who reach hospital alive, 50% die before aortic repair can be performed. 1 2 3 This high mortality rate explains why our high-volume tertiary cardiovascular surgery center observed so few BTAI cases over a period of 10 years. Besides, patients were referred for the sole purpose of aortic intervention, so we did not encounter many patients with minimal aortic injuries. Our study population included only one (7.7%) patient with grade-I aortic injury, whereas a recent multicenter retrospective study which gathered data from nine level-1 trauma centers reported 24.6% of BTAI patients had grade-I injuries. 1 Of note, this patient was treated nonoperatively, and developed a late concomitant aortic pseudoaneurysm and coarctation very similar to a case previously reported. 15

All-cause in-hospital mortality among our study population was 15.4 with 7.7% being aortic-related. These results are consistent with large multicenter retrospective studies. 1 6 16 The only patient who died primarily of aortic injury was referred from a trauma center with a grade-IV isthmus injury. The 43-year-old male arrived in extremis with cardiac tamponade. His GCS was 3, and blood pressure was 60/30 mm Hg. An emergency sternothoracotomy was performed because appropriate stent graft could not be made immediately available. The duration between his initial presentation to the trauma center and emergency operation in our center reached 11 hours. This patient's prognosis could have been better if the aortic intervention was performed at the initial trauma center. Unfortunately, most trauma centers in our country were not equipped with endovascular capabilities as of 2019. Considering the high mortality rate associated with BTAI, more trauma centers need to adapt endovascular approaches.

Among patients treated primarily with TEVAR ( *n* = 10), device-related complications occurred in one (10%). The 37-year-old motorcycle collision victim presented with aortic isthmus pseudoaneurysm, severe head injury, and left hemothorax. Aortic diameter proximal to injury was 22.1 mm. A 26 mm × 150 mm endograft was implanted obtaining adequate proximal and distal seal. LSA origin was not covered. Free rupture occurred at the postoperative hour 8, manifesting as hemodynamic collapse and excessive bleeding through the chest tube which was previously inserted for hemothorax. An emergent replacement of the descending aorta was performed through a left posterolateral thoracotomy using left heart bypass. He was discharged to home care with sequelae from head injury. This patient, along with the previously mentioned patient who underwent salvage open repair, demonstrates the fact that every center performing endovascular repair for BTAI should be experienced in open cardiovascular techniques.

Procedure-related stroke and paraplegia rates (7.7 and 0%, respectively) in the present study are similar to previous reports. 1 6 10 16 17 18 19 The only patient with a postoperative stroke was the patient who underwent Bentall's procedure. 20 We did not observe postoperative stroke or paraplegia following TEVAR in our series. Due to the natural location of isthmus injuries, a considerable number of patients need LSA coverage during TEVAR. While others report lower rates of 30 to 40%, coverage of LSA origin was needed in 70% (7/10) of our TEVAR patients. 11 21 None of these patients developed subclavian steal syndrome, posterior stroke or left arm ischemia. We perform routine imaging to assess vertebral artery dominance and the circle of Willis in our center. Clinical practice guidelines of the SVS suggest selective revascularization of the LSA. Demetriades and colleagues 6 reported only two cases who needed carotid to subclavian artery bypass among their experience of 125 BTAI patients treated with TEVAR.

Delayed repair of BTAI has been shown to improve mortality. 5 9 18 22 23 We performed delayed repair in six patients and early repair in another six. One patient died in each group. Mean ISS of delayed repair group was 28.8 (range, 16–36) versus 35.2 (range, 26–48) in the early repair group. All patients in the delayed repair group had grade-II injuries, whereas all patients in the early repair group had grade III and IV injuries.

## Conclusion

BTAI is a rare but highly fatal condition. TEVAR offers good early and midterm results for the treatment of BTAI. LSA coverage can be performed without major complications.
